# Women’s Experiences in the Process of Illness and Care During Tuberculosis Treatment: Systematic Review of a Qualitative Evidence

**DOI:** 10.3390/ijerph23010018

**Published:** 2025-12-22

**Authors:** Ana Luíza Brasileiro Nato Marques Assumpção, Flávia Correia Silva, Licia Kellen de Almeida Andrade, Quézia Rosa Ferreira, Gilberto da Cruz Leal, Mônica Cristina Ribeiro Alexandre d’Auria de Lima, Isabela Zaccaro Rigolin, Júlia Jéssica de Moraes, José Nildo de Barros Silva Junior, Rubia Laine de Paula Andrade-Gonçalves, Mônica Maria de Jesus Silva, Pedro Fredemir Palha, Jaqueline Garcia de Almeida Ballestero

**Affiliations:** Ribeirão Preto College of Nursing, University of São Paulo, Ribeirão Preto 14040-902, SP, Brazil; fcorreia@usp.br (F.C.S.); liciaandrade@usp.br (L.K.d.A.A.); quezia@usp.br (Q.R.F.); gilbertoleal@usp.br (G.d.C.L.); mcraadl@gmail.com (M.C.R.A.d.d.L.); isabelaz.rigolin@usp.br (I.Z.R.); julia.jjmoraes@usp.br (J.J.d.M.); jose.nildo@usp.br (J.N.d.B.S.J.); rubia@eerp.usp.br (R.L.d.P.A.-G.); monicamjs@usp.br (M.M.d.J.S.); palha@eerp.usp.br (P.F.P.); jaqueline.almeida@usp.br (J.G.d.A.B.)

**Keywords:** women, tuberculosis, gender perspective, qualitative approach, systematic review

## Abstract

This study aimed to identify scientific evidence that addresses women’s experiences in the process of illness and care during tuberculosis treatment. A systematic review of qualitative studies was conducted based on the Joanna Briggs Institute’s Manual for Evidence Synthesis and the Preferred Reporting Items for Systematic Review and Meta-Analysis (PRISMA) guidelines. Searches were performed in the Web of Science, MEDLINE, Embase, SciVerse Scopus, and LILACS databases. The following descriptors were used: “Women,” “Tuberculosis,” “Gender Perspective,” and “Qualitative Approach.” Studies reporting the experiences of women with active tuberculosis, published in any language, without time restrictions, were included. Of the 16,029 studies initially identified, 7079 duplicates were removed and 8895 were excluded after title and abstract screening. A total of 55 studies were read in full, of which 41 did not meet the eligibility criteria, resulting in a final inclusion of 14 studies. Most selected studies were conducted in high-tuberculosis-burden, low- and middle-income countries between 2000 and 2024. All studies focused on pulmonary tuberculosis, with one addressing drug-resistant tuberculosis. The results revealed that women’s experiences with tuberculosis are shaped by the following four thematic categories: (1) stigma and social vulnerability of women with tuberculosis; (2) gender factors in access to health services and in the interruption of tuberculosis treatment among women; (3) intersectionality and social determinants of health; and (4) the importance of social, family, and healthcare support in the experience of tuberculosis illness. The experience of illness due to tuberculosis among women is profoundly shaped by vulnerabilities related to gender, stigma, and social class, which intensify social exclusion, hinder access to diagnosis and treatment, and deepen physical and emotional suffering. Stigma reinforces isolation and weakens support networks, while the burden of domestic labor, caregiving responsibilities, and economic dependence makes it difficult for women to prioritize their own health. In this context, family support and gender-sensitive, humanized healthcare are essential. Therefore, the implementation of public policies that address these specificities and confront structural inequalities is urgent to ensure comprehensive care and a dignified, effective recovery for women with tuberculosis.

## 1. Introduction

Globally, in 2023, approximately 10.8 million people were diagnosed with tuberculosis (TB), of whom 3.6 million were women aged 15 years or older, representing 33% of the total estimated cases [1]. Although TB has a higher prevalence among men, it remains the leading cause of death from a single infectious agent among women. In the same year, 32% of TB deaths among HIV-negative individuals occurred in women, while 36% of TB-related deaths were among women living with HIV [1,2].

The literature indicates that TB treatment is often perceived by women as an additional challenge, particularly by those who are socially assigned the role of caregivers within their families, who must manage the dual burden of domestic and professional work, as well as the stigma associated with the disease. From this perspective, TB-related interventions gain particular importance when gender-specific dimensions of illness and care are taken into account [3].

Early identification of TB cases among women, improved access to diagnosis and treatment, appropriate management of comorbidities (such as HIV coinfection), and adequate psychosocial support are crucial measures to strengthen TB control from a gender-informed perspective [3].

Therefore, it is essential to recognize and address the social, economic, and cultural barriers faced by women with TB, as these factors directly influence treatment adherence. Such an approach requires acknowledging the biopsychosocial dimensions of care and challenging the patriarchal logic that continues to confine women’s health to the maternal–child domain [4].

In pursuit of global transformation, the Sustainable Development Goals (SDGs) established key targets such as Goal 3.3, which aims to end TB as a public health problem by 2030, and Goal 5, which emphasizes gender equality as a central pillar in building more equitable societies [5].

It is important to note that many studies remain limited to epidemiological comparisons between sexes and fail to explore the social and cultural implications of gender. Furthermore, they rarely examine how gender-based social constructs influence individuals’ engagement with TB diagnosis and treatment. This gap undeniably constrains the development of more effective strategies to combat the disease, particularly in vulnerable contexts [6].

Previous reviews have examined gender-related disparities in tuberculosis diagnosis and treatment, highlighting how social and structural factors shape access to care. For example, Storla (2008) [7], in a systematic review of 58 studies, identified female sex, poverty, stigma, and barriers to healthcare access as key factors contributing to delays in diagnosis and treatment. However, these contributions were predominantly epidemiological in nature and did not investigate the subjective meanings and lived experiences underlying these patterns. Building on this earlier work, the present review advances the field by synthesizing qualitative evidence focused specifically on women’s lived experiences of illness and care, and by applying an explicit intersectional gender lens to understand how social determinants shape these trajectories.

Based on these considerations, this study aimed to identify scientific evidence that addresses women’s experiences in the process of illness and care during TB treatment.

## 2. Methods

### 2.1. Study Design

This study is a systematic review of qualitative studies, conducted in accordance with the Joanna Briggs Institute (JBI) Manual for Evidence Synthesis (Chapter 3, Systematic Review of Qualitative Evidence) and the Preferred Reporting Items for Systematic Reviews and Meta-Analyses (PRISMA) guidelines [8,9]. The PRISMA Checklist is available in the supplementary document.

Qualitative research is generally undertaken to achieve a deeper understanding of a given phenomenon from the perceptions of those involved, emphasizing subjective dimensions rather than focusing on numerical or statistical quantification or the generalization of findings [10].

For the development of this review, the JBI guidelines were followed through nine steps: 1. Formulation of the review question; 2. Development and registration of the review protocol; 3. Definition of eligibility criteria; 4. Design of the search strategy; 5. Selection of scientific literature; 6. Critical appraisal of study quality; 7. Data extraction; 8. Data synthesis and evaluation of the quality of evidence; and 9. Dissemination of the results [8].

### 2.2. Review Question and Protocol Development/Registration

The review question was formulated using the PICo framework, in which P (Population) refers to women with tuberculosis (TB) and I (Phenomenon of Interest) refers to the experiences in the process of illness and care during treatment. The Co (Context) was not specified in this study, as the authors opted for a broad range of contexts. Thus, the guiding question was: “What are women’s experiences in the process of illness and care during TB treatment?”

It is noteworthy that data collection commenced only after the review protocol had been developed, registered, and published in the International Prospective Register of Systematic Reviews (PROSPERO) [11]. The registration number is CRD420250651369.

### 2.3. Eligibility Criteria

To select primary studies, the following eligibility criteria were applied: qualitative studies reporting on the experiences of women with drug-sensitive or drug-resistant TB, emphasizing specific aspects of illness and care across different stages, including diagnosis, treatment follow-up and/or recovery; and studies conducted in diverse contexts such as healthcare services, prisons, and municipal, state, or national settings.

Studies that incorporated a gender perspective into their analyses were included, particularly those that used gender as an analytical category. This approach enabled a deeper understanding of the specific trajectories of women navigating illness and receiving care. This criterion was operationalized through a careful reading of the study objectives, methods, and results to determine whether gender was approached as a structuring element of the experiences analyzed. Specifically, studies were included when they discussed socially attributed roles, unequal access to resources, power relations, or intersections with other social markers such as class and race/ethnicity. Thus, only studies in which gender was used as an interpretive category and not merely as a sociodemographic variable were considered eligible.

Exclusion criteria were: studies that addressed TB care in women from the perspective of men or healthcare professionals, studies that did not use gender as an analytical category, studies employing mixed methods, or those not available in full text. Duplicate records and documents that did not meet the criteria regarding study type or population were also excluded.

Therefore, excluded studies included: those examining TB care from the perspective of healthcare professionals or male patients, studies in which women were not the analytical population focus, mixed-methods research, theses, dissertations, and undergraduate final projects. These academic works were excluded because they are not subject to peer review, a process that ensures the scientific rigor and reliability of the publications included. Preference was given to peer-reviewed articles published in indexed journals, thereby ensuring methodological rigor, standardization of data presentation, and accessibility to the scientific community.

### 2.4. Search Strategy

The descriptors and keywords “Women,” “Tuberculosis,” “Gender Perspective,” and “Qualitative Approach” were identified and selected using the Health Sciences Descriptors (DeCS), Medical Subject Headings (MeSH), and preliminary literature searches aimed at expanding the synonym base. Boolean operators AND were used to narrow and refine the search, while OR was employed to maximize the retrieval of relevant articles, with necessary adaptations made for each specific database (Table 1).

The bibliographic search was carried out in December 2024 across the following databases: Web of Science, MEDLINE (Medical Literature Analysis and Retrieval System Online), CINAHL (Cumulative Index to Nursing and Allied Health Literature), Embase (Excerpta Medica Database), Scopus (SciVerse Scopus), and LILACS (Latin American and Caribbean Health Sciences Literature). No restrictions were applied regarding the language of publication or the date of publication.

### 2.5. Study Selection, Data Extraction and Data Analysis

After completing the search, the data were exported into Rayyan QCRI, a web-based platform developed by the Qatar Computing Research Institute. This tool supported and facilitated the organization and management of the review process, including the identification and removal of duplicate records, as well as the screening and selection of eligible studies [12].

This initial screening was performed independently by two researchers. A third reviewer was consulted to resolve disagreements or uncertainties regarding study inclusion or exclusion.

Studies selected during this stage underwent full-text assessment, which allowed for a more detailed evaluation of their relevance and eligibility for inclusion in the review. At this point, reasons for the exclusion of each document were systematically documented. The entire search and selection process was conducted in accordance with the Preferred Reporting Items for Systematic Reviews and Meta-Analyses (PRISMA) guidelines and was illustrated using the PRISMA flow diagram [8].

Data extraction was performed using a standardized form developed based on items recommended by the Joanna Briggs Institute (JBI) for systematic reviews of qualitative evidence. The information extracted from each included study consisted of: Authors; Year of publication; Journal name; Methodology; Phenomenon of interest; Study setting; Population; Data analysis approach; Authors’ conclusions; Reviewer comments. Finally, thematic analysis was employed to organize, synthesize, and present the emergent categories [13].

The synthesis of findings followed the meta-aggregation approach recommended by the Joanna Briggs Institute (JBI). After data extraction, two reviewers independently read and coded the qualitative findings from each study, including participant quotations and author-interpreted results. Through iterative comparison and discussion, these codes were grouped into categories based on similarities in meaning and content, following the principles of qualitative content analysis. These categories were subsequently synthesized into overarching analytical themes that addressed the review question, capturing the main dimensions of women’s experiences of illness and care in the context of tuberculosis.

### 2.6. Researcher Reflexivity and Positionality

Our review team comprised researchers with professional experience in tuberculosis care, qualitative methods, and gender studies. We recognize that our prior theoretical orientation, namely, that gender norms and intersecting social determinants influence health experiences, could shape how evidence is interpreted. To enhance rigor and reduce bias, we implemented reflexive strategies throughout: (i) independent dual screening and data extraction; (ii) line-by-line coding by two reviewers with iterative memo-writing; (iii) documentation of analytic decisions (audit trail) and regular consensus meetings with a third reviewer; and (iv) attention to disconfirming/negative cases during synthesis. These procedures sought to balance theoretical sensitivity with openness to findings as reported in the primary studies.

### 2.7. Theoretical and Analytical Framework

This review was guided by an analytical framework grounded in gender theory, which conceptualizes gender as a social and structural determinant that shapes both the experience of illness and access to healthcare [14].

According to Scott [14], gender is not merely a biological distinction but rather a constitutive element of social relations grounded in perceived differences between the sexes and, crucially, a primary way of giving meaning to power relations. Moving beyond a merely descriptive approach, this perspective enables a deeper analysis of how social norms, symbols, and cultural practices construct masculinities and femininities, which directly shape how individuals live, work, and interact at all levels, including their engagement with the health system and their trajectories of care [15,16].

In the field of public health, Morgan et al. [15] emphasize that operationalizing gender as an analytical category requires recognizing it as a structuring axis of social inequalities, rather than merely treating it as a descriptive variable. Incorporating this analytical lens into health systems research therefore supports the development of more strategic, effective, and equitable programs and public policies.

Moreover, understanding gender as an analytical category acknowledges that distinctions between women and men in the health–disease continuum are not limited to biological factors. They are mediated by social, cultural, historical, and political determinants. Gender, therefore, constitutes a relational and dynamic category that structures inequalities, shapes roles, norms, and gender expectations, and influences access to healthcare, experiences within health systems, and health outcomes [16].

Considering the premise that social identities cannot be understood in isolation or through a one-dimensional lens, as they are multiple, interdependent and intersecting [17], an intersectional approach was adopted. As proposed by Crenshaw [18], intersectionality is a conceptual framework that seeks to explain how different systems of oppression, such as racism, patriarchy, class-based oppression, and other mechanisms of exclusion, interact to produce vulnerabilities and establish asymmetries that shape the positions of women, races, ethnicities, classes, and other social groups. Furthermore, this analytical perspective exposes how institutional practices and public policies can perpetuate and reinforce oppression along these axes, resulting in dynamic processes of marginalization and disempowerment.

These concepts were not merely used as theoretical references but also served as analytical lenses that guided the interpretation of the findings, enabling the identification of gender-related and structural determinants throughout the literature.

In the context of TB, this approach is essential for revealing how socially constructed gender roles, conditions of dependency, differentiated stigma, and interactions with the health system are shaped by gender dynamics. These factors influence every stage of the illness trajectory from the recognition of symptoms to treatment adherence and the redefinition of life after illness.

Therefore, the use of this theoretical and analytical framework is justified, as examining women’s experiences with TB through an intersectional lens, considering gender, race/ethnicity, class, and other social markers, aims not only to describe the unique challenges faced by each woman or to simply compare men and women, but also to challenge and broaden the ways in which public health understands TB, care, and health outcomes. Ultimately, it seeks to uncover the power structures and social constructions that shape women’s experiences with TB.

### 2.8. Methodological Quality Appraisal

The methodological quality of the studies included in this review was evaluated using the qualitative research appraisal checklist proposed by the Joanna Briggs Institute [19].

## 3. Results

**Figure 1:**
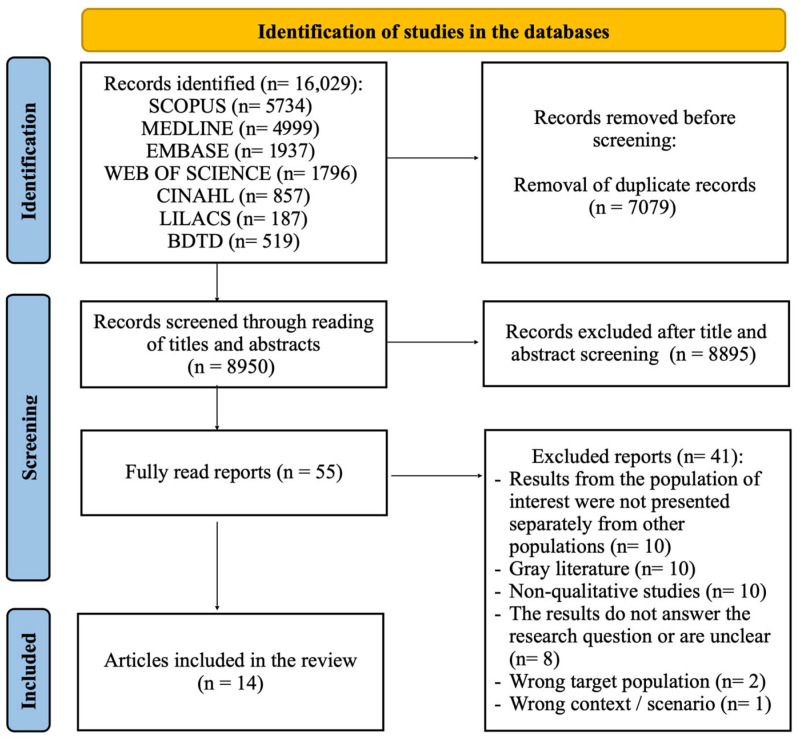
Flowchart of Study Selection Included in the Systematic Review: Women’s Experiences of Illness and Care in the Context of Tuberculosis, Brazil, 2025. Source: Adapted from Page et al. [9].

A total of 16,029 studies were identified. Of these, 7079 were excluded as duplicates. After screening the titles and abstracts of the remaining 8950 records, 55 articles were selected for full-text assessment. Following the application of the eligibility criteria, 41 studies were excluded. Consequently, 14 studies met the inclusion criteria and were included in this review (Figure 1).

The studies included in this review were published between 2000 and 2024, with one article published in 2000 [20], one in 2008 [21], two in 2010 [22,23], two in 2012 [24,25], one in 2015 [26], one in 2019 [27], one in 2020 [28], one in 2022 [29], one in 2023 [30], and three in 2024 [31,32,33].

Regarding the study locations, one was conducted in Vietnam [20], one in Peru [22], one in Brazil [23], one in the Philippines [25], one in Colombia [26], one in South Africa [30], two in Uganda [31,32] and one in Indonesia [33]. Three studies were conducted in India, with two exclusively in that country [24,28] and one with a multicenter design [21], also carried out in Bangladesh and Malawi. Two other studies also had multicentric designs: one was conducted across four countries in Eastern Europe and Central Asia—Georgia, Kazakhstan, the Republic of Moldova, and Tajikistan [29], and the other in Bangladesh, Nepal, and Pakistan [27].

As for clinical characteristics, all studies focused on pulmonary TB [20,21,22,23,24,25,26,27,28,29,30,31,32,33]. With regard to the resistance profile, only one study [31] specifically addressed drug-resistant tuberculosis (DR-TB), while the remaining studies focused on drug-sensitive TB. Details on the data collection methods and main findings of each study are presented in Table 2.

Regarding the methodological quality of the included studies (Table 3), all studies [20,21,22,23,24,25,26,27,28,29,30,31,32,33] demonstrated strong congruence between: the stated philosophical perspective and the research methodology, between the research methodology and the research question or objectives, between the research methodology and the data collection methods, and between the research methodology and the representation and analysis of data. Furthermore, participants and their voices were adequately and faithfully represented across all studies [20,21,22,23,24,25,26,27,28,29,30,31,32,33]. Finally, the conclusions presented in the research reports were appropriately derived from the data analysis and interpretation in all studies [20,21,22,23,24,25,26,27,28,29,30,31,32,33].

A statement locating the researcher culturally or theoretically was explicitly present in eight studies [20,22,23,24,25,28,29,32]; absent in three studies [26,27,33]; and unclear in three studies [21,27]. The researcher’s influence on the research and vice versa was addressed in five studies [20,22,23,29,32]; not addressed in three studies [21,31,33]; and unclear in five studies [24,25,26,27,28,30]. Regarding ethical considerations, 11 studies provided evidence of ethical approval granted by an appropriate ethics committee or institutional review board [20,22,23,25,26,27,28,29,30,32,33]; such evidence was not present in 2 studies [21,24]; and it was unclear in 1 study [31].

All studies included in this review were conducted in countries with a high TB burden, predominantly in low- and middle-income settings, reflecting the global concentration of research on women’s experiences with TB in these contexts.

## 4. Discussion

As proposed, this review sought to address a gap in the literature by synthesizing qualitative evidence on women’s experiences with TB, thereby revealing the specificities of the illness and care process. The findings demonstrate that the experience of TB among women is intrinsically shaped by social and cultural factors and is strongly influenced by gender roles, which intersect with broader social determinants of health. The following discussion aims to deepen the analysis of these findings by contextualizing them with epidemiological evidence and highlighting their implications for the development of more equitable and gender-sensitive approaches to care.

The studies were grouped into categories based on thematic analysis: 1. Stigma and social vulnerability of women with TB; 2. Gender factors in access to health services and in the interruption of TB treatment among women; 3. Intersectionality and social determinants of health; and 4. The importance of social, family, and healthcare support in the experience of TB illness.

It is important to emphasize that, although the categories are presented separately for analytical and didactic clarity, the gender dimensions that shape the illness and care experiences of women with TB are not compartmentalized in reality. Instead, the categories discussed here coexist, overlap, and interact in complex and dynamic ways, contributing to the construction and reinforcement of specific vulnerabilities.

Moreover, the organization of these categories reflects the main themes that emerged from the studies included in this review. We acknowledge that gender inequalities, along with other forms of intersecting oppression, can act as significant barriers to both accessing and remaining within health services. In many cases, women and individuals with dissident gender identities face situations of neglect, discrimination, and stigmatization, often being forced to endure such forms of violence simply to secure even minimal access to healthcare [35].

### 4.1. Stigma and Social Vulnerability of Women with Tuberculosis

Although stigma associated with TB is a factor that affects all genders indiscriminately, evidence from the studies included in this review, as well as from the broader literature, indicate that it plays a particularly structuring role in women’s illness experiences. More than an individual emotional response, stigma operates as a powerful mechanism of social exclusion, intertwined with determinants such as poverty, gender, motherhood, marital status, and access to information [36].

Therefore, the results of this review [20,23,25,30,31,32,33] reveal that stigma is neither an isolated nor merely symbolic phenomenon, but rather a historical and social construct that intensifies the vulnerability of women with TB and undermines their access to care throughout all stages of the therapeutic process.

Stigma emerged as a pervasive barrier across contexts, shaping women’s trajectories from diagnosis to treatment. In studies conducted in Uganda [31,32], social stigma hindered disclosure of the diagnosis and heightened social isolation and emotional suffering. Women reported being excluded from family and community life, experiencing rejection, separation of utensils, and restrictions on their participation in social events due to fears of contagion. In many cases, the association of TB with HIV further exacerbated stigma, compounding discrimination and deepening marginalization.

Fear of rejection and misinformation were also central in a Brazilian study [23], where TB continued to be associated with death and HIV/AIDS. This misconception, rooted in limited access to accurate health information, aggravated emotional suffering and revealed the urgent need for biopsychosocial support and educational interventions as part of comprehensive care strategies.

Similar dynamics were observed in other contexts [20,25], where fear of discrimination and feelings of shame discouraged women from seeking healthcare services. Participants reported concealing their illness from family members and neighbors out of fear of abandonment or rejection, the loss of emotional bonds, and even missed opportunities for marriage. In urban poverty settings, where women already faced financial insecurity and heavy family responsibilities, stigma emerged as a silencing force that restricted their autonomy in health decision-making.

Traditional gender roles, which assign women a subordinate position in the family hierarchy, further influenced the impact of stigma. A study conducted in Indonesia [33] showed that treatment interruption was not perceived as individual negligence but rather as a consequence of women’s double workload, subordination to male authority, and the low social value attributed to their health, particularly when it is not tied to reproductive issues. Gender-based prejudice contributed directly to neglect, blame, and distancing from healthcare services, illustrating how stigma is embedded in patriarchal structures.

Pregnancy amplified these vulnerabilities. A study [30] conducted in South Africa found that despite general knowledge about TB, pregnant women continued to face stigma, misinformation about TB–HIV coinfection, and fears of social exposure during prenatal care. This resulted in delayed healthcare-seeking, increased maternal and neonatal morbidity, and intensified psychological suffering. Stigma weighed particularly heavily on pregnant bodies, historically regulated and disciplined by both moral and biomedical norms.

Taken together, the evidence shows that TB-related stigma directed toward women is far from an individual or subjective issue. It is a socially produced phenomenon, sustained by structural inequalities and gender norms that position women in states of relational, economic, and symbolic vulnerability. Confronting this stigma requires health policies that move beyond the biomedical model and incorporate intersectoral actions in education, communication, and the empowerment of women recognizing their trajectories, strengths, and forms of resistance throughout the care process.

These findings are consistent with global evidence showing that TB-related stigma disproportionately affects women, leading to social isolation, reduced community support, and psychological distress [36,37,38]. By synthesizing qualitative evidence, this review deepens that understanding and highlights the need for gender-sensitive, community-based interventions.

### 4.2. Gender Factors in Access to Health Services and in the Interruption of Tuberculosis Treatment Among Women

The search for and access to healthcare services, as well as adherence to TB treatment from a gender perspective, are imbued with symbolic, social, structural, and emotional meanings, and therefore cannot be analyzed solely in biomedical terms. Gender roles profoundly shape how the disease is perceived and managed, influencing attitudes toward seeking healthcare and continuing or completing treatment [39].

In this review, studies [21,26,31,32,33] examine how gender-related factors influence women’s search for diagnosis and continuity of care during TB, revealing how social determinants intersect with gender to create structural barriers throughout the illness and care process.

A Colombian study [26] highlighted how social constructions of gender deeply shape the experience of illness due to TB, as well as how men and women interpret, experience, and respond to diagnosis and treatment. The authors found that women often tend to minimize physical symptoms, which contributes to delays in seeking diagnosis and accessing care. However, women were also more likely to report the emotional and psychological impacts of illness, including sadness, shame, psychological distress, and fear of social rejection.

According to Guspianto [33], gender roles strongly influence longitudinal care and contribute to women’s difficulties in accessing services and continuing TB treatment. The study demonstrated how gender dynamics operate as barriers to therapeutic adherence on social, biological, and psychological levels. For women, TB was experienced through the fear of discrimination and rejection by family and community, compounded by social isolation and a persistent sense of inadequacy arising from the challenges of fulfilling multiple roles, as mothers, wives, workers, and self-care agents. Thus, the burden of socially constructed gender norms shapes women’s tendency to prioritize family care over self-care, thereby contributing to treatment interruption.

Study [31] reinforced how the lived experiences of women undergoing treatment for MDR-TB affect both treatment adherence and coping. Participants reported that diagnosis was accompanied by shock, anxiety, fear, and feelings of hopelessness, particularly due to the perception of MDR-TB as a resistant, highly stigmatized, and often fatal condition. Additionally, mental and emotional burden was intensified by their caregiving roles, compounded by economic hardship and the physical dependency resulting from the debilitating effects of the disease.

Lack of knowledge about the disease was identified as a central factor contributing to diagnostic delays, uncertainty about transmissibility, and feelings of insecurity during treatment [23,26]. Practical barriers in the diagnostic process also significantly contributed to delaying care. In particular, difficulties in performing specific tests—such as sputum collection—emerged as a major obstacle, posing particular challenges for women [20].

Patrimonial violence, highlighted in some studies [28,33], was identified as a significant barrier to accessing health services and contributed to treatment interruption. Many women were financially dependent on their partners, and when these men refused to provide funds for treatment or transportation, their access to care was compromised or discontinued.

In study [32], conducted in Uganda, women were found to face specific challenges across the TB care continuum due to the intersection of stigma, economic dependency, and patriarchal norms that limit their autonomy to seek help. A multicenter study [21] conducted in Bangladesh, India, and Malawi observed that the decision to initiate and maintain treatment often does not rest solely with the women but depends on permission or support from male figures, highlighting a scenario of social and health subordination.

The studies and their findings reinforce the importance of recognizing gender-specific needs in care practices in order to provide psychosocial, financial, and community support particularly for women in heightened vulnerable situations. Therefore, understanding healthcare-seeking behaviors and therapeutic continuity through a gender-sensitive lens requires a broad and intersectional perspective that also considers the social determinants shaping women’s experiences with TB and the specificities of illness and care.

### 4.3. Intersectionality and Social Determinants of Health

Intersectionality provides a powerful analytical lens for examining and explaining the multiple dimensions of the complexity of the world, people, and their experiences. It is grounded in the understanding that different social markers such as gender, race/ethnicity, social class, generation, among others, intersect in mutually constitutive ways to shape social positions, power dynamics, and lived experiences, thereby producing and amplifying inequalities [18].

In this sense, applying this framework to the field of health, Bowleg [17] argues that recognizing the existence of multiple intersecting identities is fundamental to understanding not just the presence but the nature and degree of health inequities among historically oppressed groups. Beyond serving as an analytical perspective, intersectionality offers a critical framework for public health, transforming how structural determinants are understood as interconnected systems of oppression and redefining how these inequities and inequalities are conceptualized, addressed and ultimately challenged in practice [17].

Similarly, Hankivsky [40] illustrates how health research can be redefined through an intersectional approach. This approach overcomes a binary view of gender to assess health outcomes interconnected with other social determinants, especially historical and geographic determinants, providing unique health experiences.

Understanding tuberculosis in women requires moving beyond a biomedical framework toward deeply political and social analysis, as the disease itself, and the experiences surrounding it, are profoundly shaped by intersecting social determinants. Incorporating an intersectional perspective into this analysis is essential in the field of public health, as it enables a deeper understanding of how different social markers such as gender, class, race, territory, migration, and age operate in an interconnected and inseparable ways to produce experiences that heighten health disparities, exacerbate vulnerabilities, and hinder access to healthcare [35].

These intersecting forces are neither random nor isolated; rather, they operate as what Paul Farmer [41] defines as structural violence, deeply rooted social, political, and economic structures, including poverty, sexism, racism, and institutional neglect, that structure and perpetuate inequality. Structural violence becomes embodied as unequal risks of illness, suffering, and death, shaping the lived experiences of women with TB and limiting their opportunities for health and well-being.

Across the included studies, [19,21,24,30,31] emphasize how the process of illness in women with TB is strongly connected to historical, social, cultural structures and shaped by overlapping systems of oppression. These findings underscore that care trajectories are not neutral but structured by power relations that sustain inequality across multiple dimensions.

As Muttamba et al. [32] identified, intersecting factors with gender affect the process of diagnosis, treatment, and care for women with TB. According to the women in the study, determinants such as age, class, relationship status, and geographic location intersect with gender, increasing risk and vulnerability. Older women, widows, those living in poverty, and those from rural areas reported greater obstacles in facing TB. These experiences illustrate that social and health determinants do not function in isolation, but rather form a system of overlapping vulnerabilities that impact risk, suffering, and access to healthcare.

Evidence from a multicenter study [27] showed that cultural characteristics and social organization, along with intersections between gender, social class, territory, and culture, intensified the impact of TB on women’s bodies, especially in South Asia. In this context, TB not only threatens physical health but also undermines prospects for marriage, family life, and social inclusion. It is perceived as a moral failure, disqualifying women from fulfilling the socially expected roles of wives, mothers, and caregivers.

The Vietnamese study [20] highlighted how barriers faced by women with TB stem from overlapping social class, territory, and educational disadvantages. Women, particularly in rural settings, with low incomes and low levels of schooling, faced severe mobility restrictions, economic dependence on husbands and in-laws, as well as the burden of domestic work and family care responsibilities. This scenario concretely illustrates what Hankivsky [40] emphasizes: the importance of framing the complexity of human life and social inequalities by taking into account historical and geographical determinants. In this case, belonging to rural contexts intersects with class and gender, producing unique experiences of illness and care.

Similarly, research conducted in urban slums in Peru [22] analyzed the main obstacles to TB management and showed how gender disparities, combined with poverty, social roles, and patriarchal culture, directly interfered with access to and continuity of care. In this context, the risks of TB are highly gendered, and the moral responsibility for their illness and that of their families is placed on women, but the material conditions to support care are not guaranteed. The findings demonstrate that patriarchal norms, normalized in these women’s daily lives, shape family structures, illness, and care experiences. The findings of this study align with Bowleg [17], as they demonstrate how systems of oppression, such as patriarchy, sexism, and classism, intersect at a macrostructural level to sustain asymmetries in healthcare.

The study conducted in a poor urban settlement in the Philippines by Hu et al. [25] deepened the intersectional perspective by revealing that the challenges faced by women with TB result from the complex interplay of institutional, cultural, economic, and familial factors. Belonging to a marginalized, low-income social class affected care dynamics, as mistrust in the health system, due to stigma, made seeking care a constant negotiation between invisibility and survival. Together, the evidence from Peru and the Philippines illustrates how intersectionality helps explain the persistence of health disparities: TB in women cannot be understood in isolation, but only in relation to the macrostructural systems of oppression that shape daily life and access to care.

Pregnancy and motherhood also emerged as social markers that compound the social determinants of health and intensify the vulnerabilities experienced by women with TB. A study in South Africa [24] demonstrated the relationship between TB and pregnancy, showing how motherhood amplifies the impact of illness. Being a woman and undergoing pregnancy or motherhood is not just a biological condition, it is a social position filled with responsibilities, expectations, and pressure. Illness during this life stage intensifies suffering and the insecurity of being unable to fulfill the socially assigned roles of motherhood and self-care.

Another study [31] showed that married women of reproductive age experience the social, economic, and emotional impacts of TB more intensely, as multidrug-resistant TB does not exempt them from historically assigned gender responsibilities. The overlap between illness and social expectations regarding care for children, spouses, and domestic chores increases psychological and material suffering, leading to isolation, treatment interruption, depression, and financial insecurity.

Collectively, the findings from the studies in this review validate that the social determinants of health function as an oppressive system, overlapping and mutually reinforcing one another. Intersectionality, in the context of women with TB, emerges as a vital political and analytical tool for confronting structural inequalities and health disparities. Therefore, in order to mitigate oppression based on gender and other markers that influence care, it is essential to incorporate an intersectional perspective into public policies. Practices must consider the individual as a biopsychosocial subject and be implemented in a fair, equitable, and comprehensive manner.

### 4.4. The Importance of Social, Family, and Healthcare Support in the Experience of Tuberculosis Illness

The experience of falling ill with TB is consistently shaped by a series of challenges that extend beyond the physical symptoms of the disease. Economic, emotional, and social factors deeply affect the lived experiences of those diagnosed. In this context, family and social support, as well as the establishment of trust and a welcoming attitude by health services, are essential for coping with TB throughout the illness and for ensuring adherence to treatment.

Studies [23,24,31] show that support networks, both emotional and institutional, mitigate the effects of stigma, loneliness, and material hardship, significantly contributing to a successful care trajectory. Thus, for care to be more comprehensive, humane, and sensitive to the vulnerabilities experienced by women with TB, it is essential to understand the value of these forms of support.

A study conducted in Brazil [23] revealed that, particularly for women with TB, social, family, and healthcare support are fundamental elements in their experience of confronting the disease. Participants emphasized that beyond the perception of clinical improvement, a qualified welcoming approach and an effective bond with healthcare professionals, especially nurses and community health workers, were decisive factors for continuing treatment.

The study also highlighted greater social and emotional burdens among single mothers and female heads of households, due to lack of family support and precarious employment ties, which generated more difficulty in coping with the illness and, consequently, hindered treatment adherence [23].

Neoliberalism impacts the economic reality of women, imposing conditions that directly affect their quality of life and health outcomes. The rise in the number of women who are the sole financial providers for their families, coupled with unequal wages, the undervaluation of female labor, and the triple burden of work, are all key factors that shape their vulnerability [35].

At the same time, the fragility of welfare incentives, such as delays in the distribution of food baskets and transportation vouchers, was perceived as a barrier, especially for those in situations of socioeconomic vulnerability. These findings reinforce the need to strengthen community and social support networks, alongside gender-sensitive healthcare practices, to ensure treatment adherence, improve quality of life, and increase treatment effectiveness for women experiencing TB [23].

An Indian study [24] further emphasized the impact of gender roles on the illness and care process for women with TB. The absence of support networks was identified as a critical factor that hindered continuity of care. The data clearly showed that women’s traditional role as caregivers, rather than as individuals in need of care, made the illness experience more difficult. Participants reported feeling obligated to accompany children, husbands, and other relatives to healthcare services when they were ill, but not receiving the same support themselves when sick. This asymmetry made their own experience of illness and care invisible and neglected [33].

A study involving women with multidrug-resistant TB [31] reported that when family support was present, it was a crucial factor in managing suffering, ensuring access to food, transportation to appointments, and fostering treatment adherence. Women who received emotional, financial, and practical support from family networks reported a greater capacity to cope with the long and complex treatment process for MDR-TB.

Ultimately, the experience of falling ill with TB goes far beyond the disease itself, being profoundly shaped by economic, emotional, and social challenges. In this scenario, family and social support, combined with qualified care and strong patient–provider relationships, serve as critical pillars for confronting TB and promoting treatment adherence, especially among women. Support networks, whether affective or institutional, are essential for reducing the impacts of stigma, loneliness, and material difficulties, thereby contributing to more effective care processes and better quality of life.

The studies included in this review reaffirm that the presence of such support is a key differentiator, with the empathy and commitment of healthcare professionals playing a decisive role in treatment continuity. However, in the absence of such support, socioeconomic vulnerabilities and gender roles can increase the burden on women with TB and hinder their care journey. To achieve comprehensive and humane care, it is essential to strengthen these support networks and implement healthcare practices that are sensitive to gender and vulnerability issues, thereby ensuring a more effective and equitable response to TB.

Finally, the qualitative evidence synthesized in this review demonstrates that women’s experiences of illness and care are deeply shaped by structural determinants such as poverty, gender norms, educational inequities, and unequal access to resources. These determinants operate beyond the individual level, producing and sustaining the conditions in which stigma, delayed diagnosis, and barriers to treatment adherence persist. This recognition aligns with global perspectives that view gender inequality as a fundamental driver of health inequities. Sen and Östlin [42] emphasize that gender-based power imbalances and structural discrimination must be addressed to achieve equitable health outcomes. Similarly, the World Health Organization’s Gender Assessment Toolkit [43] calls for the incorporation of gender-responsive approaches in TB policies and programs to confront these systemic barriers. By contextualizing women’s lived experiences within these broader frameworks, this review underscores the need for intersectoral actions that move beyond the biomedical model, integrating social, economic, and cultural interventions to reduce disparities and strengthen the responsiveness of TB services to women’s specific needs.

## 5. Policy and Practice Implications

### 5.1. Empowerment and Agency of Women in TB Care

The findings of this review reveal that women’s limited autonomy and decision-making power significantly constrain their ability to seek timely diagnosis, adhere to treatment, and navigate the healthcare system. Addressing these barriers requires interventions that go beyond clinical care and actively promote women’s empowerment and agency throughout the tuberculosis care continuum. Community-based initiatives that engage women as partners in program design and delivery, peer support groups that foster mutual learning and resilience, and collaborations with women’s organizations in TB awareness campaigns can strengthen women’s voices and participation in care. Furthermore, future qualitative research and program evaluations should involve women not merely as study participants but as key stakeholders contributing to the co-creation of solutions. Placing women’s perspectives and leadership at the center is crucial to building health interventions that are both gender-responsive and contextually grounded.

### 5.2. Healthcare Provider Training in Gender-Sensitive Care

Several findings underscore the central role that healthcare delivery practices play in shaping women’s experiences of TB care. A qualified, welcoming approach, trust-building, and respect for confidentiality emerged as key factors influencing women’s willingness to seek and remain in care. These insights point to the need for systematic training of healthcare providers, including nurses, physicians, and community health workers, in gender-sensitive and culturally competent care. Such training should address how gender norms shape barriers to access, promote communication that reduces stigma and fosters trust, and incorporate practical measures such as flexible scheduling to accommodate women’s caregiving responsibilities. As emphasized by Ghosh [44], cultural competence and gender sensitivity are essential for creating inclusive healthcare environments and ensuring equitable access to services.

### 5.3. Integration of Psychosocial and Social Support Interventions

This review also highlights the pivotal role of social and family support in women’s treatment experiences and outcomes. Policies and programs should integrate psychosocial support into TB care, including counseling services, peer support groups, and partnerships with social services and non-governmental organizations. These collaborations can help provide material support, such as transportation vouchers, food packages, and childcare assistance during clinic visits, which is often critical for treatment adherence. Special attention should be given to single mothers and female-headed households, who face heightened vulnerability to poor treatment outcomes due to intersecting social and economic disadvantages. Strengthening the social protection components of TB programs can substantially reduce these inequities and improve overall care outcomes.

### 5.4. Community Engagement and Stigma Reduction

Given the pervasive impact of stigma documented in this review, interventions must extend beyond the clinical setting to address stigma at the community level. Community education and engagement strategies that challenge gendered stereotypes and misinformation about TB are essential. Partnering with women’s organizations, local leaders, and community-based groups can amplify messages that counter stigmatizing narratives, for instance, dispelling the notion that women with TB are “unfit” for marriage or motherhood. In high-burden settings, TB literacy campaigns should explicitly integrate gender-sensitive messages and encourage supportive community responses. Reducing stigma through community engagement is not only a matter of social justice but also a critical determinant of improved health-seeking behavior and treatment adherence.

### 5.5. Monitoring and Intersectional Use of Data

Finally, strengthening TB surveillance and program evaluation through intersectional data analysis is essential to uncover and address hidden disparities. While sex-disaggregated data are fundamental, programs must also analyze outcomes by intersecting variables such as age, socioeconomic status, and geographic location to identify subgroups of women at greater risk of diagnostic delay, treatment interruption, or poor outcomes. As Rowley and Mugala [45] argue, without intersectional data analysis, significant inequities may remain obscured. For example, aggregate data may suggest similar treatment success rates between men and women, yet disaggregated analysis could reveal lower success among young rural women due to the barriers identified in this review. Refining monitoring systems to capture these nuances will enable TB programs to design more targeted and effective interventions.

Taken together, these implications demonstrate how a gender and intersectionality-informed approach can transform TB programs, policies, and research agendas, ultimately advancing equity and improving outcomes for women affected by tuberculosis.

## 6. Study Limitations

This review has several limitations that should be acknowledged. First, the inclusion of fourteen qualitative studies reflects not a shortcoming of this review but rather the current state of research on women and tuberculosis, which remains underexplored in the global literature. This limited number of studies is closely linked to epidemiological patterns: because women represent a smaller proportion of TB cases worldwide compared to men, research on their experiences has historically received less attention. Consequently, certain dimensions of women’s experiences may not yet be fully captured. Notably, no qualitative studies were identified on genital tuberculosis in women, a condition associated with infertility and significant social implications. This represents an important gap in the literature that future research should address.

Additionally, by restricting inclusion to studies that explicitly analyzed gender, we may have missed relevant insights reported in broader TB research. However, this methodological decision ensured that the synthesized evidence remained conceptually aligned with the objective of this review, which was to explore how gender shapes women’s experiences of illness and care.

The heterogeneity of the included studies, conducted across different countries, healthcare systems, and sociocultural contexts, is both a strength and a limitation. While this diversity enriches the synthesis by capturing a range of perspectives, it also limits the universal generalizability of the findings, as certain themes, such as stigma or restrictions on women’s mobility, are likely to be deeply shaped by local cultural norms.

Another limitation is the absence of studies focusing on transgender women and gender-diverse populations. Although this perspective was part of the initial conceptual scope of the review, the search strategy did not include descriptors specific to these groups, resulting in their exclusion. This gap highlights an important direction for future research, which should explicitly integrate gender diversity to expand the understanding of tuberculosis care experiences.

Finally, limitations inherent to qualitative evidence synthesis must be considered. Our findings rely on the data and analyses reported by the primary study authors, meaning that any methodological constraints present in those studies, such as small sample sizes, sampling biases, or contextual limitations, may carry over into this review.

Moreover, the thematic synthesis process involves a degree of interpretive subjectivity, which is both a limitation and an intrinsic feature of qualitative research. Recognizing these limitations strengthens the reflexivity and transparency of this review and underscores the need for future research, including comparative qualitative analyses across diverse sociocultural and territorial contexts, as well as studies examining how gender, race, class, sexual orientation, territory, and gender identity intersect to shape the experience of tuberculosis illness and care in both high- and low-income settings.

## 7. Conclusions

The experience of illness due to TB among women is shaped by vulnerabilities related to gender, stigma, and social class, which intensify social exclusion, hinder access to diagnosis and treatment, and deepen physical and emotional suffering. Stigma reinforces isolation and weakens support networks, while the burden of domestic work, caregiving responsibilities, and economic dependence makes it difficult for women to care for their own health. In this context, family support and gender-sensitive, humanized healthcare are essential. Therefore, the implementation of public policies that address these specificities and confront structural inequalities is urgent to ensure comprehensive care and a dignified, effective recovery for women with TB.

## Figures and Tables

**Table 1:** Search strategies according to each database.

Database	Search Strategy	Records Identified
MEDLINE	(“womans”[All Fields] OR “women”[MeSH Terms] OR “women”[All Fields] OR “woman”[All Fields] OR “women s”[All Fields] OR “womens”[All Fields] OR (“women”[MeSH Terms] OR “women”[All Fields] OR “girl”[All Fields]) OR (“girl s”[All Fields] OR “women”[MeSH Terms] OR “women”[All Fields] OR “girls”[All Fields]) OR (“womans”[All Fields] OR “women”[MeSH Terms] OR “women”[All Fields] OR “woman”[All Fields] OR “women s”[All Fields] OR “womens”[All Fields]) OR (“femal”[All Fields] OR “female”[MeSH Terms] OR “female”[All Fields] OR “females”[All Fields] OR “female s”[All Fields] OR “femals”[All Fields]) OR (“gender identity”[MeSH Terms] OR (“gender”[All Fields] AND “identity”[All Fields]) OR “gender identity”[All Fields] OR “gendered”[All Fields] OR “gender s”[All Fields] OR “gendering”[All Fields] OR “genderized”[All Fields] OR “genders”[All Fields] OR “sex”[MeSH Terms] OR “sex”[All Fields] OR “gender”[All Fields])) AND (“tuberculosi”[All Fields] OR “tuberculosis”[MeSH Terms] OR “tuberculosis”[All Fields] OR “tuberculoses”[All Fields] OR “tuberculosis s”[All Fields] OR “TB”[All Fields] OR (“tuberculosis, pulmonary”[MeSH Terms] OR (“tuberculosis”[All Fields] AND “pulmonary”[All Fields]) OR “pulmonary tuberculosis”[All Fields] OR “phthisis”[All Fields]) OR “Koch Disease”[All Fields] OR “Koch’s Disease”[All Fields] OR “Kochs Disease”[All Fields]) AND (“Qualitative study”[All Fields] OR “Qualitative approach”[All Fields] OR (“anthropology, cultural”[MeSH Terms] OR (“anthropology”[All Fields] AND “cultural”[All Fields]) OR “cultural anthropology”[All Fields] OR “ethnographies”[All Fields] OR “ethnography”[All Fields]) OR “Discourse analysis”[All Fields] OR “Content analysis”[All Fields] OR “Thematic analysis”[All Fields] OR “Social representations”[All Fields] OR (“experience”[All Fields] OR “experience s”[All Fields] OR “experiences”[All Fields]) OR (“perspective”[All Fields] OR “perspective s”[All Fields] OR “perspectives”[All Fields]) OR “Action research”[All Fields])	4999
Web of Science	Women OR Girl OR Girls OR Woman OR Female OR Gender (Topic) AND Tuberculosis OR TB OR Phthisis OR “Koch Disease” OR “Koch’s Disease” OR “Kochs Disease” (Topic) AND “Qualitative study” OR “Qualitative approach” OR Ethnography OR “Discourse analysis” OR “Content analysis” OR “Thematic analysis” OR “Social representations” OR Experiences OR Perspective OR “Action research” (Topic)	1796
Embase	#1 (‘women’/exp OR women OR ‘girl’/exp OR girl OR girls OR ‘woman’/exp OR woman OR ‘female’/exp OR female OR ‘gender’/exp OR gender) AND ([embase]/lim OR [preprint]/lim) #2 (‘tuberculosis’/exp OR tuberculosis OR ‘tb’/exp OR tb OR ‘phthisis’/exp OR phthisis OR ‘koch disease’ OR ‘kochs disease’) AND ([embase]/lim OR [preprint]/lim) #3 (‘qualitative study’/exp OR ‘qualitative study’ OR ‘qualitative approach’ OR ‘ethnography’/exp OR ethnography OR ‘discourse analysis’/exp OR ‘discourse analysis’ OR ‘content analysis’/exp OR ‘content analysis’ OR ‘thematic analysis’/exp OR ‘thematic analysis’ OR ‘social representations’ OR ‘experiences’/exp OR experiences OR ‘perspective’/exp OR perspective OR ‘action research’/exp OR ‘action research’) AND ([embase]/lim OR [preprint]/lim) #4 #1 AND #2 AND #3	1937
CINAHL	(Women OR Girl OR Girls OR Woman OR Female OR Gender) AND (Tuberculosis OR TB OR Phthisis OR “Koch Disease” OR “Koch’s Disease” OR “Kochs Disease”) AND (“Qualitative study” OR “Qualitative approach” OR Ethnography OR “Discourse analysis” OR “Content analysis” OR “Thematic analysis” OR “Social representations” OR Experiences OR Perspective OR “Action research”)	857
Scopus	TITLE-ABS-KEY(Women OR Girl OR Girls OR Woman OR Female OR Gender) AND TITLE-ABS-KEY(Tuberculosis OR TB OR Phthisis OR “Koch Disease” OR “Koch’s Disease” OR “Kochs Disease”) AND TITLE-ABS-KEY(“Qualitative study” OR “Qualitative approach” OR Ethnography OR “Discourse analysis” OR “Content analysis” OR “Thematic analysis” OR “Social representations” OR Experiences OR Perspective OR “Action research”)	5734
LILACS	(mulheres OR meninas OR mulher OR feminino OR gênero OR women OR girl OR girls OR woman OR female OR gender OR mujeres OR mujer OR femenino OR género OR femmes OR femme) AND (tuberculose OR tísica OR tuberculosis OR tb OR phthisis OR “Koch Disease” OR “Koch’s Disease” OR “Kochs Disease” OR tisis) AND (“Estudo qualitativo” OR “Abordagem qualitativa” OR etnografia OR “Análise de discurso” OR “Análise de conteúdo” OR “Análise temática” OR “Representações sociais” OR experiências OR perspectiva OR “Pesquisa-ação” OR “Qualitative study” OR “Qualitative approach” OR ethnography OR “Discourse analysis” OR “Content analysis” OR “Thematic analysis” OR “Social representations” OR experiences OR perspective OR “Action research” OR “estudio cualitativo” OR “Enfoque cualitativo” OR etnografía OR “Análisis del discurso” OR “Análisis de contenido” OR “Análisis temático” OR “Representaciones sociales” OR experiencias OR perspectiva OR “Investigación acción” OR “Analyse de contenu” OR “Analyse thématique” OR “Représentations sociales” OR expériences OR perspective OR “Recherche-action”) AND db:(“LILACS”) AND instance:”lilacsplus”	187

Caption: The literature searches were performed on 16 December 2024. Source: the authors.

**Table 2:** Information and synthesis of the main results of studies selected for systematic review.

ID	Author/ Year/ Journal/ Country	Study Objective	Type of Study/Place of Study/Participants	Data Collection/ Data Analysis	Main Findings
A1 [20]	Johansson et al./2000/Health Policy/Vietnam	To explore and describe perceptions of factors influencing health-seeking behavior in Vietnam, with specific reference to gender differentials in delay in health-seeking.	Qualitative study based on grounded theory. Four FGDs were conducted in four districts. The groups consisted of men with TB, men without TB, women with TB, and women without TB. Participants with personal experience of TB were purposefully selected by the researchers and the local TB team. The groups consisted of eight to ten participants and were relatively homogeneous in terms of socioeconomic status, but not in terms of age. The participants were unknown to each other.	The study conducted four focus groups in each of four Vietnamese districts, with sessions lasting one to two hours. Discussions were recorded, transcribed in Vietnamese, and translated into English. Data were analyzed inductively, with open coding applied by two independent researchers, followed by team discussions. Codes were compared across gender, urban/rural location, TB experience, and region, and reorganized into categories derived from the data.	The study found that delays in seeking care for TB were influenced by fear of social isolation, economic constraints, and poor quality of health services. Women primarily delayed care due to stigma and fear of social consequences, often using private services or self-medicating first, while men delayed mainly because of the personal costs of diagnosis and treatment, tending to ignore symptoms until the disease became severe and then seeking public services directly.
A2 [21]	Weiss et al./2008/The International Journal of Tuberculosis and Lung Disease/Bangladesh, India and Malawi	To identify and compare sociocultural aspects of TB with reference to gender, analyzing experiences, meanings and behaviors of patients in the three countries studied.	Cultural epidemiological study using qualitative and quantitative methods. Approximately 100 patients at each of three sites, with a balance between men and women and representation of patients at different stages of treatment.	Use of semi-structured interviews, categorization of patient reports, and application of non-parametric statistics to compare patterns of suffering, perceived causes, and help-seeking.	Female patients reported a wider range of symptoms, while men focused more on financial concerns. Most patients experienced psychological and emotional distress. Men attributed TB to smoking and alcohol, whereas women in Malawi linked it to sexual causes associated with HIV/AIDS. In Bangladesh, fears of disease transmission despite treatment led to social isolation of women. Preferences for care varied: public health services in Malawi and private doctors in India and Bangladesh.
A3 [22]	Onifade et al./2010/BMC Public Health/Peru	To characterize gender-related barriers to tuberculosis control in Peruvian slums using semi-structured interviews and focus group discussions with tuberculosis patients and healthcare professionals.	This qualitative study used grounded theory to explore in-depth and culturally specific knowledge about TB and gender. The intentional sample included 43 participants: 14 women, 12 men, and 17 healthcare professionals.	Reports of the health experiences of male and female TB patients and health professionals were obtained through two data collection methods: semi-structured interviews and focus group discussions.	We found that the TB program was perceived not to be gender discriminatory and provided equal TB diagnostic and treatment care to men and women. This contrasted with stereotypical gender roles in the broader community context and a commonly expressed belief amongst patients and healthcare workers that female health inherently has a lower priority than male health. This belief was principally associated with men’s predominant role in the household economy and limited employment for women in this setting. Women were also generally reported to experience the adverse psychosocial and economic consequences of TB diagnosis more than men.
A4 [23]	Queiroz et al./2010/Saúde e Sociedade/Brazil	To verify differences in adherence to TB treatment in relation to gender; to identify aspects that facilitate and hinder adherence to TB treatment in relation to gender; to analyze the beliefs considered important for adherence to TB treatment.	This qualitative study was based on the theoretical framework of Rosenstock’s Health Belief Model and Bardin’s Content Analysis technique. Twenty-eight semi-structured interviews were conducted with men and women undergoing supervised TB treatment.	Content analysis according to Bardin, structured in the categories of the Health Belief Model: perceived susceptibility, perceived seriousness, perceived benefits and perceived barriers.	Out of 13,448 studies, 28 were included, all from low- and middle-income countries. They examined individual-level barriers to TB care, with some also assessing provider/system-level barriers. Barriers affected both genders but differed in nature and impact: women faced financial and physical dependence, lower literacy, and household stigma, while men experienced work-related financial and physical barriers and community-based stigma.
A5 [24]	Khan et al./2012/Health Care for Women International/India	To understand the lived experiences and explore the issues and challenges that people with TB have faced throughout their disease journey. To share the personal stories of women living with TB from Delhi’s slums and situate these stories within the broader social contexts of these women’s lived experiences. With an intersectional focus on gender and its impacts on women’s health and the healthcare process.	This is a qualitative ethnographic study. Informal group discussions were held, with four groups composed of men and two groups of women who self-identified as TB-free. In-depth interviews were conducted with selected patients (who participated in short, structured interviews). Fifteen in-depth interviews were completed with women and ten with men.	Data were collected through informal group discussions, short, structured interviews with TB patients, and in-depth follow-up interviews, all conducted in Hindi and later translated into English. The study found that women’s suffering with TB is more closely tied to social context and life structure than to the disease itself, with experiences shaped by intersections of gender, class, caste, family support, and rural–urban dynamics, which influenced their illness and care processes.	Women’s narratives highlighted a high prevalence of stigma, reinforced by daily interactions and shaped by poverty, migration, and the sociocultural context of favela life, leading many to deny their diagnosis or delay treatment. Despite these challenges, women showed strength and resilience, developing coping strategies and forming supportive networks. Stigma disproportionately affected women, rooted in sexist perceptions that condemn them for “unexpected or unwanted actions,” resulting in greater family and community rejection and more severe consequences than for men.
A6 [25]	Hu et al./2012/Health Care for Women International/Philippines	To characterize the experiences of low-income urban women in the Philippines in seeking TB care and to identify barriers and facilitators to care, from a socioecological perspective.	Qualitative study (semi-structured interviews and focus groups). The interview participants were 13 women with TB (aged between 23 and 73, most married, most working in the informal sector); three focus groups were also conducted with mothers from the community.	Thematic analysis based on transcription of interviews and focus groups, using Atlas.ti software; coding based on emerging themes and research objectives.	Care-seeking among low-income urban women with TB is shaped by shame, stigma, financial constraints, and family responsibilities, along with barriers in the health system such as poor service quality and limited information. Support from family and community health workers facilitated care, while trajectories varied from rapid to delayed or incomplete treatment.
A7 [26]	Vélez et al./2015/Investigacíon y Educacíon en Enfermería/Colômbia	To determine gender differences in the interpretation of TB in a group of patients from the city of Medellín.	Qualitative study, using the grounded theory method. A total of six men and six women who were index cases of families followed up after treatment was completed.	Interviews followed a structured script with adjustments as needed, and data were analyzed using grounded theory through constant comparison, open coding, and the development of descriptive categories. Atlas Ti 5.1 was used to organize and support the analysis.	Cough was the most common symptom, with men emphasizing physical symptoms and women reporting emotional effects. Both genders feared infection, social rejection, and work loss, while women also experienced shame and difficulty with treatment adherence.
A8 [27]	Hatherall et al./2019/Qualitative Health Research/Bangladesh, Nepal and Pakistan	To develop an explanatory theory about the impact of TB on women’s marriage prospects in South Asian countries, based on the relationship between TB, gender roles and social stigma, using a qualitative approach grounded in a realist perspective of causality.	A primary qualitative study using Grounded Theory methodology and thematic analysis was based on 73 individual interviews and eight focus groups. The interviews were conducted with 48 people with TB (25 women and 23 men), 15 TB patients’ family members (11 women and 4 men), and 10 health professionals (6 women and 4 men).	Interviews were transcribed and translated into English. Coding and analysis were based on Grounded Theory, guided by a realist perspective on causality (the relationship between mechanisms and context). NVivo 7 software was used for data organization. Cross-validation was performed between the analyses conducted by local teams and the principal investigator.	The study found that TB threatens women’s social and familial roles, leading to stigma that can affect marriage prospects and risk of divorce. Women often hide their diagnosis, but mobility, family control, and low education limit confidential treatment access. The healthcare system and current TB policies can reinforce stigma through frequent clinic visits, confidentiality breaches, and insensitivity to gender issues.
A9 [28]	Mukerji et al./2020/Global Public Health/India	To identify the challenges perceived by female TB patients in India for successful delivery of DOTS.	For this qualitative study, focusing on TB stigma, we conducted in-depth qualitative interviews. Twenty non-pregnant female TB patients over the age of eighteen who were (1) currently taking medication or (2) had completed DOTS treatment in the past six months or more were invited to participate in this study.	Between May and June 2016, 20 interviews were conducted in Bengali or Hindi to explore women’s treatment-seeking behaviors, medication experiences, and TB stigma, mapping their journey from symptoms to DOTS initiation. Interviews were transcribed, translated, de-identified, and analyzed using Nvivo 11.3.2 with thematic coding and a socioecological framework to examine barriers at individual, interpersonal, institutional, community, and policy levels.	TB stigma in women primarily led to social isolation, gossip, verbal abuse, failed marriage prospects, and family neglect, resulting in non-disclosure, guilt, and mental health issues such as suicidal thoughts. Women coped through positive strategies like prayer, talking with peers, and relaxation, while negative strategies included self-isolation and anger. Stigma-related non-disclosure sometimes affected TB transmission and control.
A10 [29]	Turusbekova et al./2022/BMC Public Health/Georgia, Kazakhstan, Moldova, and Tajikistan	To analyze how gender-related factors are constructed to highlight the necessary improvements in the care of women with TB.	This qualitative study involved 86 interview participants and 227 participants in 21 focus groups. All participants were over 18 years old and had not been diagnosed with TB for more than five years. In total, 130 respondents were women, with the exception of Kazakhstan, where the number of men and women was equal.	The data were analyzed by the collaborating authors and their teams according to three predefined stages: symptom recognition, seeking medical care, and obtaining a correct diagnosis. Gender-related factors and barriers encountered by people with TB at each stage were recorded by the focus group interviewees and note-takers, who were provided with a form to categorize the information to facilitate analysis.	The study identified gender-related barriers affecting women’s TB symptom recognition, care-seeking, and diagnosis. Key barriers included: (1) Family and financial obligations, limiting women’s ability to travel for testing and prioritize their own health; (2) Stigma and social consequences, with married women fearing divorce and single women facing reduced marriage prospects, leading to concealment of disease; and (3) Health system barriers, such as misdiagnosis, assumptions that women are at lower risk, discomfort with sputum collection, and exposure to TB through caregiving roles.
A11 [30]	Khoza et al./2023/BMC Public Health/South Africa	To explore the educational needs of pregnant women diagnosed with TB to prevent complications during pregnancy and the neonatal period.	This qualitative descriptive study involved semi-structured individual interviews, using thematic analysis. Sixty-three pregnant women with TB participated.	Thematic analysis of transcribed data, guided by the research questions; coding, identification of themes and subthemes.	Despite pregnant women having general knowledge about TB disease, the knowledge and awareness regarding the prevention of TB complications in pregnancy and the neonatal period, information on TB/HIV and COVID-19 co-infections, and participants’ knowledge about other non-infectious diseases that may affect the mother with TB infection and fetus showed a deficit.
A12 [31]	Omona et al./2024/Cogent Public Health/Uganda	To investigate the experiences of female patients aged 15–49 years receiving treatment for MDR-TB at Lira Regional Referral Hospital, Uganda.	Qualitative phenomenological study. Fifteen female patients with MDR-TB who were of reproductive age (15–49 years) and undergoing treatment for MDR-TB at Lira Regional Referral Hospital, Uganda, participated in the study.	Data were collected using the Lango guide translated into English for in-depth interviews. Using a purposeful approach, patients who attended the health service to receive their medication were invited to participate in the study. The recorded interviews were transcribed and analyzed using NVivo version 13 software for thematic analysis.	Women with MDR-TB, particularly married and of reproductive age, experience greater mental and social suffering than men due to caregiving duties, economic precarity, and gender stigma. These pressures lead to isolation, depression, treatment interruption, and difficulties in adhering to care, while family and social support are crucial for treatment success.
A13 [32]	Muttamba et al./2024/BMC Public Health/Uganda	To identify social stratifications that interrelate with gender and influence TB treatment, in order to understand how best to develop gender-sensitive intersectional interventions to control the disease, as part of a gender-equitable program.	Cross-sectional qualitative study. Two urban health facilities in Buikwe and Kiboga districts, and two rural health facilities in Nakaseke district, central Uganda. Twenty-six key informant interviews were conducted (ninetten men and seven women).	The interviews were recorded using a digital recorder and notepad by the researchers depending on the informant’s preferences, and were analyzed through content analysis.	Women’s TB vulnerability is increased by biomass exposure from household roles, but they have more frequent health system contact through childcare. Men often prioritize work over healthcare and delay seeking care, influenced by beliefs in their strength. Women’s care-seeking decisions are often dependent on husbands or partners, making the process complex.
A14 [33]	Guspianto et al./2024/Journal of Health Research/Indonesia	To provide an overview of gender bias in the TB treatment programs.	This qualitative, descriptive–exploratory study involved interviews with eight female patients who discontinued TB treatment in the Batanghari region. To validate the data, two health professionals from the TB program at the public health center were also interviewed.	Interviews were recorded using a digital recorder and notes, depending on the informant’s preferences, and analyzed using content analysis.	Women dropped out of TB treatment due to medication negligence, side effects, and financial or psychological barriers. Gender bias, including men’s dominance in households and associated role expectations, resulted in women’s health being prioritized less, despite TB services being formally equal for men and women.

Caption: FGD—Focus Group Discussion; TB—Tuberculosis; HIV/aids—Human Immunodeficiency Virus that causes acquired immunodeficiency syndrome; DOTS—Directly Observed Treatment; MDR-TB—multidrug-resistant TB. Source: the authors.

**Table 3:** Methodological quality of articles included in the systematic review.

ID	Is There Congruence Between the Stated Philosophical Perspective and the Research Methodology?	Is There Congruence Between the Research Methodology and the Research Question or Objectives?	Is There Congruence Between the Research Methodology and the Methods Used to Collect the Data?	Is There Congruence Between the Research Methodology and the Representation and Analysis of Data?	Is There Congruence Between the Research Methodology and the Interpretation of Results?	Is There a Statement Locating the Researcher Culturally or Theoretically?	Is the Researcher’s Influence on Research and Vice versa Addressed?	Are Participants and Their Voices Adequately Represented?	Is the Research Ethical According to Current Criteria or Recent Studies, or Is There Evidence of Ethical Approval by an Appropriate Body?	Do the Conclusions Drawn in the Research Report Stem from Data Analysis or Interpretation?
A1 [21]	Y	Y	Y	Y	Y	Y	Y	Y	Y	Y
A2 [22]	Y	Y	Y	Y	Y	U	N	Y	N	Y
A3 [23]	Y	Y	Y	Y	Y	Y	Y	Y	Y	Y
A4 [24]	Y	Y	Y	Y	Y	Y	Y	Y	Y	Y
A5 [25]	Y	Y	Y	Y	Y	Y	U	Y	N	Y
A6 [26]	Y	Y	Y	Y	Y	Y	U	Y	Y	Y
A7 [27]	Y	Y	Y	Y	Y	N	U	Y	Y	Y
A8 [28]	Y	Y	Y	Y	Y	U	U	Y	Y	Y
A9 [29]	Y	Y	Y	Y	Y	Y	U	Y	Y	Y
A10 [30]	Y	Y	Y	Y	Y	Y	Y	Y	Y	Y
A11 [31]	Y	Y	Y	Y	Y	U	U	Y	Y	Y
A12 [32]	Y	Y	Y	Y	Y	N	N	Y	U	Y
A13 [33]	Y	Y	Y	Y	Y	Y	Y	Y	Y	Y
A14 [34]	Y	Y	Y	Y	Y	N	N	Y	Y	Y

Caption: Y (yes); N (no); U (unclear). Source: Based on Aromataris et al. [34].

## Data Availability

The original contributions presented in this study are included in the article/Supplementary Materials. Further inquiries can be directed to the corresponding author.

## Supplementary Materials

The following supporting information can be downloaded at: https://www.mdpi.com/article/10.3390/ijerph23010018/s1, Table S1: PRISMA 2020 Checklist.
